# Bioelectrical impedance phase angle in clinical practice: implications for prognosis in stage IIIB and IV non-small cell lung cancer

**DOI:** 10.1186/1471-2407-9-37

**Published:** 2009-01-28

**Authors:** Digant Gupta, Carolyn A Lammersfeld, Pankaj G Vashi, Jessica King, Sadie L Dahlk, James F Grutsch, Christopher G Lis

**Affiliations:** 1 Cancer Treatment Centers of America® (CTCA) at Midwestern Regional Medical Center, 2610 Sheridan Road, Zion, IL 60099, USA

## Abstract

**Background:**

A frequent manifestation of advanced lung cancer is malnutrition, timely identification and treatment of which can lead to improved patient outcomes. Bioelectrical impedance analysis (BIA) is an easy-to-use and non-invasive technique to evaluate changes in body composition and nutritional status. We investigated the prognostic role of BIA-derived phase angle in advanced non-small cell lung cancer (NSCLC).

**Methods:**

A case series of 165 stages IIIB and IV NSCLC patients treated at our center. The Kaplan Meier method was used to calculate survival. Cox proportional hazard models were constructed to evaluate the prognostic effect of phase angle, independent of stage at diagnosis and prior treatment history.

**Results:**

93 were males and 72 females. 61 had stage IIIB disease at diagnosis while 104 had stage IV. The median phase angle was 5.3 degrees (range = 2.9 – 8). Patients with phase angle <= 5.3 had a median survival of 7.6 months (95% CI: 4.7 to 9.5; n = 81), while those with > 5.3 had 12.4 months (95% CI: 10.5 to 18.7; n = 84); (p = 0.02). After adjusting for age, stage at diagnosis and prior treatment history we found that every one degree increase in phase angle was associated with a relative risk of 0.79 (95% CI: 0.64 to 0.97, P = 0.02).

**Conclusion:**

We found BIA-derived phase angle to be an independent prognostic indicator in patients with stage IIIB and IV NSCLC. Nutritional interventions targeted at improving phase angle could potentially lead to an improved survival in patients with advanced NSCLC.

## Background

Worldwide, lung cancer is the most common form of cancer, with an incidence of 1.35 million new cases per year, and 1.18 million deaths, with the highest rates in Europe and North America. Non-small cell lung cancer (NSCLC) accounts for about 80% of all lung cancers [1].

Malnutrition is a frequent manifestation in patients with advanced NSCLC and is a major contributor to morbidity and mortality [2]. Malnutrition is characterized by changes in cellular membrane integrity and alterations in fluid balance [3]. As a result, measurement of body composition is an important component of overall nutritional evaluation in cancer patients [4-6].

Historically, nutritional status has been evaluated by various objective measures, including anthropometric (e.g. weight change, arm muscle circumference, triceps skinfold thickness) and laboratory (serum albumin, transferrin assays and nitrogen balance studies) measurements. In the clinical setting, anthropometric methods are not ideal because they are time-consuming and require well-trained staff. Some of the objective measures such as serum albumin are likely to be influenced by many non-nutritional factors [7-10]. Furthermore, some objective indicators such as serum albumin have long half-lives, thus, assessing changes in the nutritional status over a short period of time is challenging. A less common tool to assess nutritional status, called Bioelectrical Impedance Analysis (BIA), can overcome some of these challenges. BIA is an easy-to-use, non-invasive, and reproducible technique to evaluate changes in body composition.

BIA has been validated for the assessment of body composition and nutritional status in a variety of patient populations including cancer [2,5,11-21]. BIA measures body component resistance (R) and capacitance (Xc) by recording a voltage drop in applied current [22]. Resistance is the restriction to the flow of an electric current, primarily related to the amount of water present in the tissues. Capacitance is the resistive effect produced by the tissue interfaces and cell membranes [23]. Capacitance causes the current to lag behind the voltage creating a phase shift, which is quantified geometrically as the angular transformation of the ratio of capacitance to resistance, or the phase angle [24].

Phase angle reflects the relative contributions of fluid (resistance) and cellular membranes (capacitance) of the human body. By definition, phase angle is positively associated with capacitance and negatively associated with resistance [24]. Lower phase angles suggest cell death or decreased cell integrity, while higher phase angles suggest large quantities of intact cell membranes [25]. Phase angle has been found to be a prognostic marker in several clinical conditions such as human immunodeficiency virus infection, liver cirrhosis, chronic obstructive pulmonary disease, hemodialysis, sepsis, lung cancer [25-30]. Previously, we had demonstrated the prognostic role of phase angle in advanced colorectal and pancreatic cancer [31,32]. We also recently demonstrated the prognostic role of phase angle in breast cancer [33]. The primary objective of this study, which builds upon our prior research work in this area, was to evaluate the association of BIA-derived phase angle with survival in patients with advanced NSCLC.

## Methods

A retrospective chart review was performed on a consecutive case series of 165 stages IIIB and IV NSCLC patients treated at Cancer Treatment Centers of America (CTCA)^® ^at Midwestern Regional Medical Center (MRMC) between January 2001 and May 2006 (this is the same time as that mentioned in our previous breast cancer manuscript). The patients were identified from the MRMC tumor registry. Only patients with a histologically confirmed diagnosis of stages IIIB and IV NSCLC were included in this study. The study was approved by the Institutional Review Board at MRMC.

Phase angle was measured using BIA at presentation to our hospital as part of the overall nutritional assessment of the patient. For a detailed description of statistical methods, please refer to our recently published manuscript on breast cancer [33]. For the purpose of univariate analysis, phase angle measurements were categorized using SPSS into 2 mutually exclusive groups with median = 5.3 as the cut-off. In our previous research on breast cancer, we had similarly categorized phase angle measurements using the median value as the cut-off [33]. For the purpose of multivariate analyses (linear Cox regression), phase angle was treated as a continuous variable.

## Results

**Figure 1:**
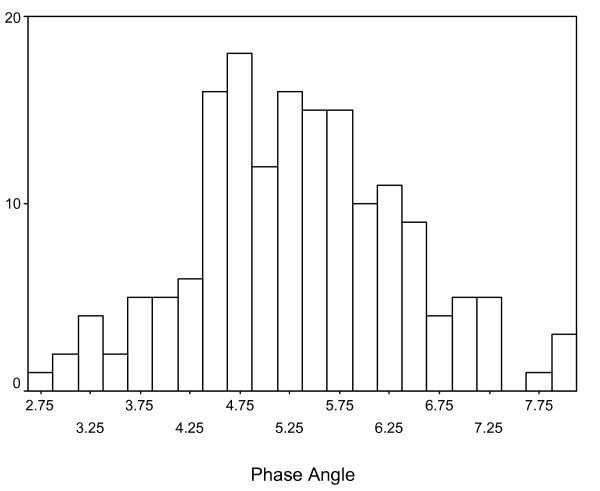
**A histogram depicting the distribution of phase angle**.

At the time of this analysis, 111 patients had expired and 54 were censored, as shown in Table 1. The median age at diagnosis was 56 years (standard deviation – 9.1 years; range 30 – 78 years). The median phase angle was 5.3 degrees (standard deviation – 1.1 degrees; range = 2.9 – 8 degrees). Figure 1 depicts a histogram showing the distribution of phase angle scores.

**Table 1:** Baseline characteristics

Characteristic	Categories	Number	Percent (%)
Sex	Male	93	56.4
	Female	72	43.6
Vital Status	Expired	111	67.3
	Censored^*1*^	54	32.7
Prior Treatment	Progressive disease	85	51.5
History	Newly diagnosed	80	48.5
Stage at Diagnosis	Stage III	61	37.0
	Stage IV	104	63.0

^*1 *^Patients who reached the end of their follow-up without experiencing death.

N = 165

Table 2 shows the univariate survival analysis of different prognostic factors. Phase angle, tumor stage and treatment history were found to be statistically significantly associated with survival while gender was not. Every one year increase in age at diagnosis was associated with a relative risk of 1.01 (95% CI: 0.98 to 1.03, P = 0.63). The mean age at diagnosis was 58.1 years (standard deviation – 8.3 years) and 53.1 years (standard deviation – 9.1 years) for the "below median" and "above median" phase angles groups respectively, the difference being statistically significant (p < 0001). Similarly, the mean phase angle in degrees was 5.6 (standard deviation – 1.1) and 4.9 (standard deviation – 0.94) for "males" and "females" respectively, the difference being statistically significant (p < 0001).

**Table 2:** Univariate Kaplan-Meier survival analysis

Variable	Survival in months	Log-rank score	P-value
Phase Angle			
• <= 5.3	7.6 (4.7 to 9.5)	6.3	0.02
• >5.3	12.4 (10.5 to 18.7)		
Gender			
• Male	8.7 (5.7 to 11.8)	2.9	0.08
• Female	12.2 (4.9 to 19.4)		
Tumor Stage			
• Stage IIIB	16.8 (9.4 to 24.3)	9.0	0.003
• Stage IV	7.7 (5.9 to 9.3)		
Treatment History			
• Newly diagnosed	14.3 (9.9 to 20.6)	9.8	0.002
• Progressive disease	6.8 (4.5 to 9.1)		

N = 165

**Figure 2:**
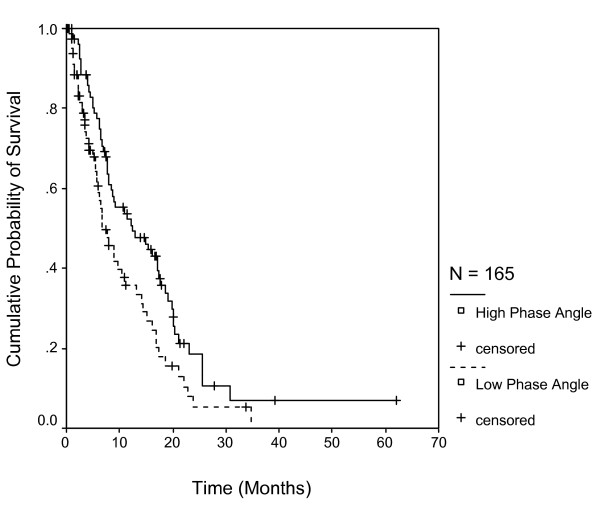
**Survival stratified by phase angle categories with cutoff of 5.3**. Each drop in a probability curve indicates one or more events in that group. Vertical lines indicate censored patients, i.e., those who reached the end of their follow-up without experiencing death.

Figure 2 shows the survival curves for the two categories of the phase angle. Patients with phase angle <= 5.3 had a median survival of 7.6 months (95% CI: 4.7 to 9.5; n = 81), while those > 5.3 had 12.4 months (95% CI: 10.5 to 18.7; n = 84); the difference being statistically significant (p = 0.02).

Table 3 summarizes the results of multivariate Cox regression analyses. Multivariate Cox modeling, after adjusting for age, stage at diagnosis and prior treatment history found that every one degree increase in phase angle was associated with a relative risk of 0.79 (95% CI: 0.64 to 0.97, P = 0.02).

**Table 3:** Multivariate Cox proportional hazard model

Independent Variable	Unit of increase	**RR**^***1***^	95% CI	P-value
Phase angle	1 degree	0.79	0.64, 0.97	0.02
Age at diagnosis	1 year	1.0	0.98, 1.02	0.99
Stage at Diagnosis	Stage IIIB as referent	1.8	1.1, 2.7	0.01
Treatment History	Newly Diagnosed as referent	1.7	1.2, 2.6	0.01

^*1 *^Relative risk (Cox proportional hazard)

N = 165

## Discussion

The identification of prognostic factors in advanced NSCLC is of considerable importance for clinical management of the disease. Tumor stage remains the single most important prognostic factor in advanced NSCLC. The current study was undertaken to investigate if BIA-derived phase angle, a potential indicator of nutritional status, could predict survival in advanced NSCLC cancer.

This study demonstrated that phase angle is a strong predictor of survival in advanced NSCLC after controlling for the effects of age, stage at diagnosis and prior treatment history. A similar study conducted in patients with advanced lung cancer stratified the patient cohort by the mean phase angle of 4.5 degrees. Interestingly, patients with phase angle less than or equal to 4.5 degrees had a significantly shorter survival than those with phase angle greater than 4.5 degrees [34]. In our previous study in stage IV colorectal cancer patients, we found that phase angle above the median cut-off of 5.6 was associated with better survival [32]. Similarly, in stage IV pancreatic cancer, phase angle above the median cut-off of 5 was associated with improved survival [31].

This study adds to the growing body of evidence regarding the clinical applications of BIA derived phase angle beyond its use in body composition equations. Although the biological meaning of phase angle is not well understood, it reflects not only body cell mass, but is also one of the best indicators of cell membrane function, related to the ratio between extracellular water and intracellular water [23]. Schwenk et al. has hypothesized that phase angle could possibly be interpreted as a global marker of malnutrition in HIV infected patients [30]. In another study conducted on HIV-infected patients, it was argued that phase angle reflects the integrity of vital cell membranes [28]. In patients with liver cirrhosis, phase angle was speculated to be a marker of clinically relevant malnutrition characterized by both increased extracellular mass and decreased body cellular mass [25]. In advanced lung cancer, phase angle was speculated to be an indicator of altered tissue electrical properties [34]. In spite of lack of standardized cut-off values, phase angle seems to play an important role as a marker of morbidity and mortality in a wide range of disease conditions, with higher phase angle reflecting a general indicator of wellness [23].

Limitations of this study relate to the BIA technique and retrospective study design. This study, because of its retrospective nature, relies on data not primarily meant for research. One potential limitation of the BIA approach for estimating body composition is the reliance on regression models, derived in restricted samples of human subjects, which limits the usefulness of the derived model in other patients who differ from the original sample in which the model was developed [35,36]. However, in our study, we looked at phase angle which does not depend on regression equations to be calculated, thereby eliminating a large source of random error [3]. It has also been suggested that the variability of direct bioimpedance measures (resistance, capacitance, and phase angle) depends on age, gender, and body mass characteristics of the study population which could possibly limit the extrapolation of the model [23,35,37]. A review article by Foster et al. argued that although the correlation between whole-body impedance measurements and body composition is experimentally well established, the reason for the success of the impedance technique is much less clear [38]. Finally, because we used linear Cox regression, there is a possibility of a floor effect with phase angle rarely, if ever, reaching much below 2 degrees. As a result, a degree difference in phase will may have a much greater relative risk between 2 and 3 degrees than 7 and 8 degrees.

The other limitations of the study are very similar to the limitations described in our breast cancer manuscript [33].

## Conclusion

In summary, our study has demonstrated the prognostic significance of phase angle in advanced NSCLC after controlling for the effects of stage at diagnosis and prior treatment history.

## Competing interests

The authors declare that they have no competing interests.

## Authors' contributions

DG was the main author of the manuscript, participated in concept, design, data collection, data analysis and data interpretation. CAL, JK, and SLD participated in concept, design, data collection and writing. PGV participated in concept, design and data interpretation. JFG assisted with the statistical analysis and data interpretation. CGL participated in concept, design, writing and data interpretation. All authors read and approved the final manuscript.

## Pre-publication history

The pre-publication history for this paper can be accessed here:

http://www.biomedcentral.com/1471-2407/9/37/prepub
